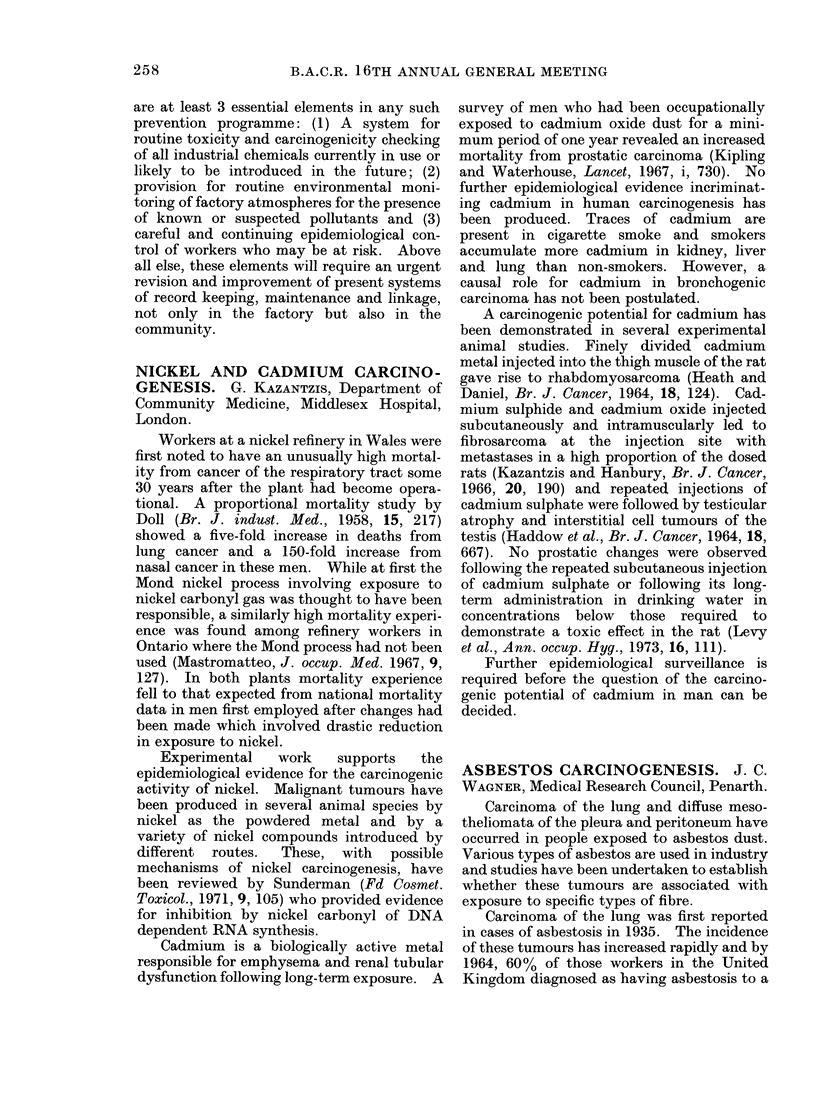# Proceedings: Nickel and cadmium carcinogenesis.

**DOI:** 10.1038/bjc.1975.205

**Published:** 1975-08

**Authors:** G. Kazantzis


**NICKEL AND CADMIUM CARCINOGENESIS.** G. KAZANTZIS, Department of Community Medicine, Middlesex Hospital, London.

Workers at a nickel refinery in Wales were first noted to have an unusually high mortality from cancer of the respiratory tract some 30 years after the plant had become operational. A proportional mortality study by Doll (*Br. J. indust. Med.*, 1958, **15**, 217) showed a five-fold increase in deaths from lung cancer and a 150-fold increase from nasal cancer in these men. While at first the Mond nickel process involving exposure to nickel carbonyl gas was thought to have been responsible, a similarly high mortality experience was found among refinery workers in Ontario where the Mond process had not been used (Mastromatteo, *J. occup. Med.* 1967, **9**, 127). In both plants mortality experience fell to that expected from national mortality data in men first employed after changes had been made which involved drastic reduction in exposure to nickel.

Experimental work supports the epidemiological evidence for the carcinogenic activity of nickel. Malignant tumours have been produced in several animal species by nickel as the powdered metal and by a variety of nickel compounds introduced by different routes. These, with possible mechanisms of nickel carcinogenesis, have been reviewed by Sunderman (*Fd Cosmet. Toxicol.*, 1971, **9**, 105) who provided evidence for inhibition by nickel carbonyl of DNA dependent RNA synthesis.

Cadmium is a biologically active metal responsible for emphysema and renal tubular dysfunction following long-term exposure. A survey of men who had been occupationally exposed to cadmium oxide dust for a minimum period of one year revealed an increased mortality from prostatic carcinoma (Kipling and Waterhouse, *Lancet*, 1967, i, 730). No further epidemiological evidence incriminating cadmium in human carcinogenesis has been produced. Traces of cadmium are present in cigarette smoke and smokers accumulate more cadmium in kidney, liver and lung than non-smokers. However, a causal role for cadmium in bronchogenic carcinoma has not been postulated.

A carcinogenic potential for cadmium has been demonstrated in several experimental animal studies. Finely divided cadmium metal injected into the thigh muscle of the rat gave rise to rhabdomyosarcoma (Heath and Daniel, *Br. J. Cancer*, 1964, **18**, 124). Cadmium sulphide and cadmium oxide injected subcutaneously and intramuscularly led to fibrosarcoma at the injection site with metastases in a high proportion of the dosed rats (Kazantzis and Hanbury, *Br. J. Cancer*, 1966, **20**, 190) and repeated injections of cadmium sulphate were followed by testicular atrophy and interstitial cell tumours of the testis (Haddow *et al.*, *Br. J. Cancer*, 1964, **18**, 667). No prostatic changes were observed following the repeated subcutaneous injection of cadmium sulphate or following its long-term administration in drinking water in concentrations below those required to demonstrate a toxic effect in the rat (Levy *et al.*, *Ann. occup. Hyg.*, 1973, **16**, 111).

Further epidemiological surveillance is required before the question of the carcinogenic potential of cadmium in man can be decided.